# Stochastic Simulation Service: Bridging the Gap between the Computational Expert and the Biologist

**DOI:** 10.1371/journal.pcbi.1005220

**Published:** 2016-12-08

**Authors:** Brian Drawert, Andreas Hellander, Ben Bales, Debjani Banerjee, Giovanni Bellesia, Bernie J. Daigle, Geoffrey Douglas, Mengyuan Gu, Anand Gupta, Stefan Hellander, Chris Horuk, Dibyendu Nath, Aviral Takkar, Sheng Wu, Per Lötstedt, Chandra Krintz, Linda R. Petzold

**Affiliations:** 1 Department of Computer Science, University of California, Santa Barbara, Santa Barbara, California, United States of America; 2 Department of Information Technology, Division of Scientific Computing, Uppsala University, Uppsala, Sweden; 3 Department of Mechanical Engineering, University of California, Santa Barbara, Santa Barbara, California, United States of America; 4 Departments of Biological Sciences and Computer Science, The University of Memphis, Memphis, Tennessee, United States of America; UCSD, UNITED STATES

## Abstract

We present StochSS: Stochastic Simulation as a Service, an integrated development environment for modeling and simulation of both deterministic and discrete stochastic biochemical systems in up to three dimensions. An easy to use graphical user interface enables researchers to quickly develop and simulate a biological model on a desktop or laptop, which can then be expanded to incorporate increasing levels of complexity. StochSS features state-of-the-art simulation engines. As the demand for computational power increases, StochSS can seamlessly scale computing resources in the cloud. In addition, StochSS can be deployed as a multi-user software environment where collaborators share computational resources and exchange models via a public model repository. We demonstrate the capabilities and ease of use of StochSS with an example of model development and simulation at increasing levels of complexity.

This is a *PLOS Computational Biology* Software paper.

## Introduction

A striking outcome of the past decade of Systems Biology research is the insight that stochasticity plays an important role in many biological processes. Examples include endogenous [1, 2] and synthetically constructed [3, 4] bistable genetic switches, noise enhanced robustness of oscillations [5, 6], and fluctuation enhanced sensitivity [7]. In many cellular systems, low local species populations can create stochastic effects even if total cellular levels are high [8]. It has been noted that numerous cellular phenomena, including polarization and chemotaxis, rely on spatial stochastic noise for robust performance [9]. Additional examples include end-to-end oscillations in MinCDE in *E. coli* [10], spontaneous polarization of *S. cerevisiae* [11], and actin mediated directed transport [12].

Discrete stochastic simulation is now an important and widely-used tool for modeling of biological systems at the molecular scale. A typical modeling project might progress from simple deterministic ordinary differential equation (ODE) models, to well-mixed discrete stochastic models, all the way to detailed spatial stochastic models, as the questions and hypotheses are refined based on increased understanding of the biological system under study. Typically, as a model becomes more complex, not only is there a steeper learning curve for the modeler to become fluent with the relevant software, but the algorithms, implementations and computer hardware required to simulate the model in wall-clock times that are useful for model development and the productive interaction between modeling and experiment explode in their complexity. The amount of computational power needed for conducting large-scale computational experiments calls for distributed computing on clusters, grids or clouds, which requires a level of computer science expertise not possessed by most biologists.

We have addressed these issues in StochSS, first by providing an integrated development environment powered by an easy-to-use web-based user interface (WebUI) that allows model transition from the simplest ODE models to the most complex spatial stochastic models, backed by native 2D and 3D visualization for smooth model debugging and presentation, and second by making it simple to scale computational resources as the problem size grows without the need to integrate with complicated distributed systems. This is achieved via cloud computing. With the push of a button, StochSS expands computing capabilities by deploying the computations to the public Amazon EC2 cloud. Models in StochSS can be built with the WebUI, or alternatively can be imported directly into the model editor in either StochKit XML [13] or SBML [14] format.

Taken together, StochSS provides an integrated solution that addresses model specification on multiple levels, features state-of-the-art simulation algorithms for efficient simulation, and removes the barrier of scaling out computational resources when needed.

StochSS employs a hierarchy of quantitative models of reaction kinetics and the associated software to study the dynamics of cellular reaction networks and related problems. On the deterministic level, where the system is governed by a set of ODEs (the rate equations), StochSS makes use of the CVODES solver for stiff systems of ODEs from the well-known SUNDIALS package [15]. On the stochastic well-mixed level, where the distribution of molecules is assumed to be spatially homogeneous, StochSS offers the Stochastic Simulation Algorithm (SSA) [16, 17] (commonly known as the Gillespie method) as well as adaptive time-stepping tau-leaping [18], as implemented in the StochKit2 [13] stochastic simulation package for well-mixed systems. For spatial stochastic simulation in up to 3 dimensions, StochSS offers highly efficient implementations of the Next Subvolume Method (NSM) [19] and the Adaptive Diffusive Finite State Projection (ADFSP) algorithm [20, 21], as implemented in the PyURDME [22] spatial stochastic simulation package. The meshes are unstructured, and the spatial geometries can be complicated and may include multiple domains, for example membrane and cytoplasm. Mesoscale algorithms model the diffusion of molecules by a discrete jump process on a computational mesh. Reactions occur with a given probability when molecules occupy the same voxel in the mesh. They are very efficient compared to their microscale (particle-tracking) counterparts. For a review of the most commonly used algorithms and their computational and theoretical background, see e.g. [23].

A number of software packages support model building and simulation at one or several of the scales outlined above. COPASI [24] and Tellurium [25], implement well-mixed and deterministic methods. PySB [26] is a Python package for rule-based model development with well-mixed simulations. MCell [27], Smoldyn [28], and Readdy [29] feature microscale, particle-tracking algorithms for spatial stochastic simulation. STEPS [30] and MesoRD [31] support mesoscopic spatial stochastic simulations; STEPS on unstructured meshes and MesoRD on structured meshes. In contrast to the above software packages, StochSS features capabilities at the deterministic well-mixed level, the stochastic well-mixed level, and the mesoscopic spatially inhomogeneous level, but does not yet support the more fine-grained, but also more expensive, microscale simulations. StochSS also supports sensitivity analysis at the deterministic level (via SUNDIALS [15]) and parameter estimation for well-mixed stochastic systems via [32].

Like StochSS, E-Cell [33] and Vcell [34] feature a range of methods at different scales. StochSS has additional functionality compared to E-Cell and VCell, such as scaling of computational resources to the cloud. E-Cell and StochSS implement a spatial stochastic mesoscopic solver, while VCell does not. Both VCell and StochSS have multi-user functionality and offer the possibility of sharing models through a public model repository, but StochSS can be downloaded and installed on a private server or laptop while VCell requires that the user connect to a central server to be functional.

Some of the software packages listed above offer capabilities in addition to model building and simulation: COPASI has functionality for deterministic parameter estimation, local sensitivity analysis, and linear noise approximations; VCell offers parameter estimation through COPASI, supports simple import of models from biomodels.net, lets the user define initial conditions using experimental fluorescence imaging data, and supports parameter sweeps; MCell has the capability of advanced graphics rendering via Blender.

While several software packages are currently available, together offering numerous computational tools, StochSS offers much of that functionality in an integrated software package. With a web based GUI and the possibility of scaling simulations to the cloud, StochSS has been developed with the aim to make modeling and simulation easier for the user. With the capability to be deployed as Software as a Service (SaaS) on a privately owned server or in cloud Infrastructure as a Service (IaaS), StochSS aims at reducing the barriers of collaborative modeling between members in a team by providing a joint environment for computational experimentation. This feature also makes StochSS a powerful tool for educational efforts in quantitative modeling, since it enables use of StochSS on popular and cheap clients.

## Design and Implementation

**Fig 1:**
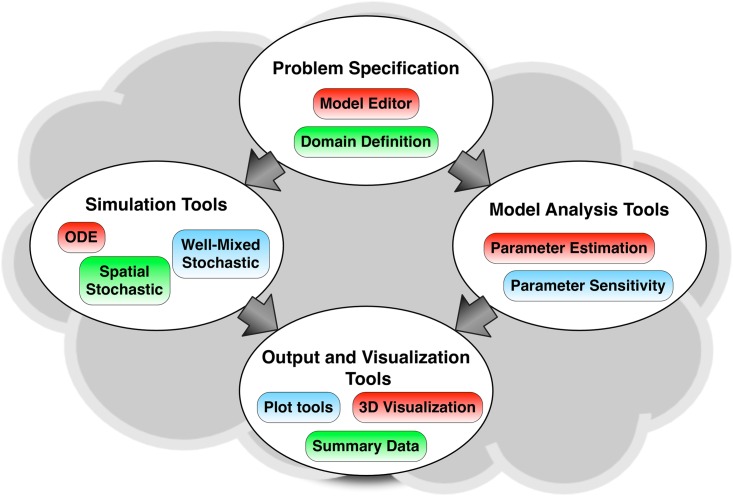
Process flow and component diagram for a modeling and simulation workflow with StochSS. The biochemical model and domain is defined as part of the problem specification. The ODE, spatial stochastic, and well-mixed simulation tools generate realizations of these models. The parameter estimation and parameter sensitivity tools allow for analysis of models. The output and visualization tools present the data.

The conceptual structure and range of capabilities of StochSS is illustrated in Fig 1. StochSS features a collection of tools designed for the modeling and simulation of well-mixed chemically reacting models, discrete stochastic models, and spatial stochastic models, exposed to the user through a simple, powerful, cross-platform WebUI. A broad array of state-of-the-art simulation engines, for deterministic models, well-mixed discrete stochastic models, and spatial stochastic models on complex geometries, are available. StochSS also features tools for the analysis of models, including tools for sensitivity analysis (for ODE models), and for parameter estimation for discrete stochastic models. Model outputs can be obtained as graphs, and in the case of spatial simulation, by solid and volume rendering with animation. In this section we provide an overview of model specification, simulation, analysis, and output and visualization in StochSS.

### Model Specification

**Fig 2:**
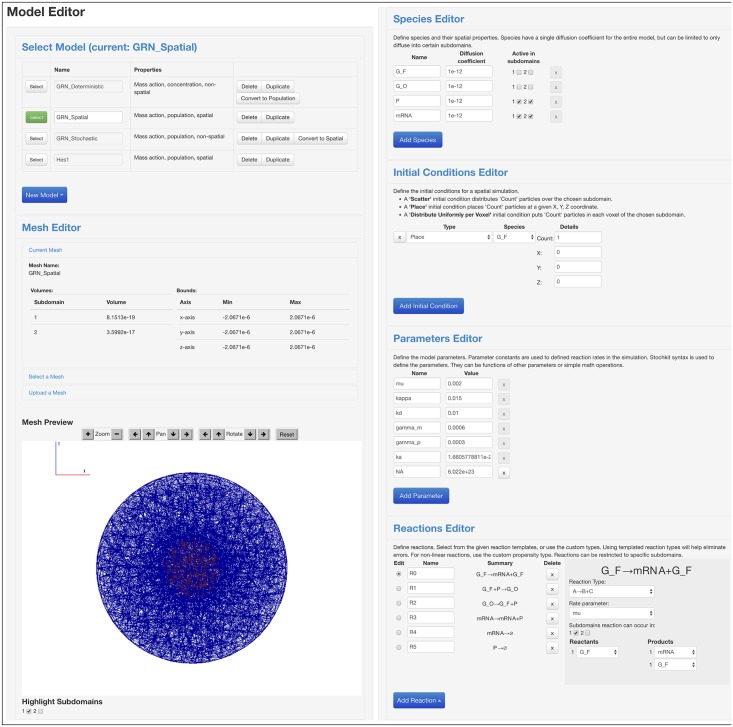
Screenshots of the StochSS model editor. Choose the model to edit from the selection list and view and edit your domain with the mesh editor (left), and define the biochemical species, initial conditions, parameters, and reactions (right).

One of the most powerful features of StochSS is the Model Editor (shown in Fig 2), which allows for simple specification of a biochemical model. Model specification begins by defining the *species*, *parameters*, and *reactions* of the biochemical system, as well as the initial conditions (either concentration of population) for each species. The model can be specified as either deterministic (concentration based) or discrete stochastic (population based). The StochSS Model Editor features the capability to easily transition between types of models. For example, well-mixed concentration models can be converted to well-mixed discrete models. In this process, the user inputs a system’s volume, after which the conversion of species and mass-action reactions is done automatically. Such an automated transformation is only possible with mass-action reactions. For models with custom propensities, the conversion tool will prompt the user to assist in the proper conversion of the propensity functions.

StochSS users are not required to build their models inside StochSS; models defined elsewhere in either Stochkit XML or SBML format can be imported directly in the model editor. If an SBML model has features not supported by StochSS simulators, such as multiple compartments in well-mixed models, the model editor will import what it can and then issue warnings about which SBML feature (such as SBML functions, events or compartments) was ignored so that the user can resolve any problems. SBML import/export is only available for well mixed models due to the fact that there are to date no fully developed standard for expressing spatial models.

In addition to well-mixed models, StochSS supports discrete stochastic reaction-diffusion models for spatially inhomogeneous biochemical systems. In these models, the user must specify a tetrahedral 3D mesh (standard meshes in e.g. Finite-Element (FE) computations), the diffusion coefficient for each species, and initial spatial distribution of the species. Users may also specify sub-domains, or regions within the mesh (e.g. the cytoplasm and nucleus of a cell) and restrict reactions and species to specific parts of the geometry. StochSS provides simple conversion from population based well-mixed models to spatial models. To help expedite 3D model development, some sample meshes are included in the system so that experimental models can be developed without having to deal with importing a custom mesh. A custom mesh can be imported into StochSS in the FEniCS/Dolfin [35] XML mesh format, and a tool will create StochSS-compatible meshes from Gmsh [36], a popular open source mesh generator. A tutorial for how to create and then import an externally defined mesh into a StochSS model is available on the StochSS website and in S1 Text.

### Simulation and Analysis Tools

Once a model is created, the next step is to perform simulations of the model with the *Simulation Manager* tool. The user simply selects the model to simulate and is then presented with the possible simulation types that are supported for that particular model. For example, for a well-mixed concentration based model, the user can select between ‘Deterministic’ and ‘Deterministic + Sensitivity’. Well-mixed population based models have those options and the additional option of ‘Stochastic’ simulation (with SSA or Tau-leaping). The user then specifies the end time for the simulation, the sampling interval, and any other input relevant to that simulation type, such as which parameters to compute sensitivities for, or the number of trajectories in the stochastic ensembles.

Once the simulation is set up, it is submitted for execution via either the ‘Run Local’ or ‘Run Cloud’ button. The actual computation is handled by one of four simulation tools, depending on which type of simulation has been requested. The CVODES solvers from the Sundials software package [15] are used for ODE simulation with the backward differentiation formula (BDF) linear multistep method as well as for sensitivity analysis. Discrete stochastic simulation of well-mixed chemically reacting systems is accomplished via our StochKit2 [13] software package. The StochKit2 package includes the popular Gillespie Stochastic Simulation Algorithm (SSA) direct method [16, 17], Optimized Direct Method (ODM) [37], and the constant-time SSA [38], and automatically selects between these algorithms. It also includes the adaptive non-negativity preserving explicit tau-leaping method [18]. Spatial stochastic simulation is accomplished via our PyURDME [22, 39] package. PyURDME is a framework for modeling and simulation of stochastic reaction-diffusion processes on unstructured, tetrahedral (3D) and triangular (2D) meshes. Unstructured meshes allow for a more flexible handling of complex geometries compared to structured, Cartesian meshes. The current core simulation algorithm that is exposed to the StochSS user is based on the mesoscopic reaction-diffusion master equation (RDME) model [40] and is an optimized implementation of the next subvolume method (NSM) [19] from the URDME software [39].

Finally, our StochOptim software enables the estimation of parameters for well-mixed discrete stochastic systems, using time-series data. Parameter estimation is accomplished via the Monte Carlo Expectation-Maximization with Modified Cross-Entropy Method (MCEM^2^) algorithm [32]. This method requires no prior knowledge of the parameter values, and automatically provides a multivariate parameter uncertainty estimate. To perform the parameter estimation in StochSS, the user inputs an initial guess for the parameters in the model editor and uploads observed time-series data. StochOptim then performs an iterative computation to match the stochastic simulation trajectories to those of the input data file. The computation concludes when convergence is detected, however the user is able to stop the computation at any point and create a new model with the currently estimated parameters. A tutorial and format specification for the data is available as part of the StochSS manual, available on the StochSS website and in S1 Text.

### Output and Visualization Tools

**Fig 3:**
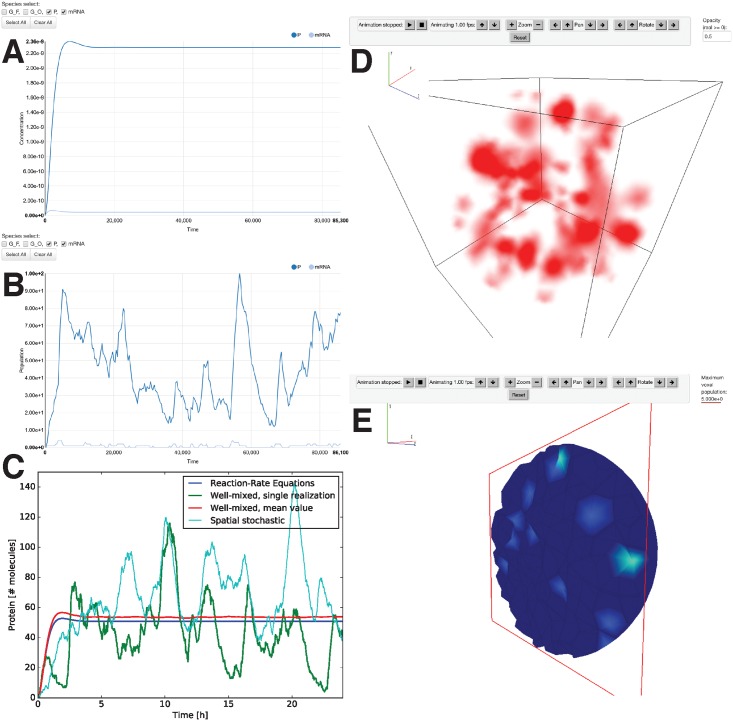
StochSS provides built-in visualization capabilities in order to quickly explore simulation results such as deterministic ODE simulations (A) and well-mixed discrete stochastic realizations (B). Using external plotting libraries, in this case matplotlib in Python, we highlight the key qualitative differences between the deterministic and stochastic simulations (C). As can be seen, while the mean values differ slightly between the model levels, the most dramatic difference is apparent when considering individual realizations, which reveals a high noise expression level of transcription factor. For spatial stochastic modeling, the model editor provides the capability to visualize the computational mesh and the subdomains as wireframes (see Fig 2), and simulations can be visualized and animated, in this case using volume rendering (D) and solid rendering with domain clipping (E).

Visualization and analysis of simulation results is an important part of the model development process. We have developed a suite of visualization tools for StochSS that make the viewing of data produced by its internal tools quick and easy. For well-mixed systems, StochSS supports time-series plots for visualization of trajectories using the real-time HTML5 plotting library NVD3.js, which is based on D3.js [41]. Fig 3A and 3B show samples of the plotting. The user may select and plot different species and trajectories dynamically, allowing for rapid exploration of a model’s behavior.

StochSS also features a set of 3D visualization tools for animating and exploring the time-series volumetric data that is the output from the spatial solvers. The processing of 3D spatial simulation datasets can be both complex and time-consuming, so it is important for StochSS to include its own easy-to-use internal visualization system. There are two popular ways of rendering 3D information: a raster technique and a ray-tracing technique. The raster technique is implemented by rendering the model mesh using WebGL [42], and coloring the voxel faces (using a jet colormap) based on concentration values of the selected biochemical species interpolated between the nearby vertices. Fig 3E show examples of visualization of surfaces and cross-sections of a spatial model. The raster technique presents concentration information only on surfaces. To visualize the internal system dynamics with this technique, we provide a domain slicing feature whereby the user can visualize internal cross-sections of the 3D volume, see Fig 3E.

The second 3D rendering tool in StochSS is a ray-tracing volume renderer [43]. Volume renderings make it easy to visualize structure in the concentration fields produced in spatial stochastic simulation. A 3D volume visualization enables users to view a succinct picture of the entire state of their simulations. A volume rendering of a spatial model is shown in Fig 3D. Animation is enabled for both of these techniques, allowing users to visualize the temporal dynamics of biochemical systems.

For complete analysis of a simulation result, it is often necessary to export the dataset to an external programming environment for specialized analysis. StochSS enables the export of simulation data in raw data format. The format of the simulation data depends on the underlying tool used to perform the simulation. For well-mixed simulation results, the data is exported as a zip file containing a set of flat text files in the StochKit2 format. For spatial simulations, the user can export the data as the native PyURDME result object, or as a zip file containing a set of CSV files: one to show the spatial information for the mesh and one for time-series data of each simulation trajectory. In addition, if further visualization and analysis is required for spatial simulation results, spatial data can also be exported in VTK format for use in Paraview [44] or any other compatible program.

### Cloud Computing Capabilities

One major of the features of StochSS is its capability to utilize cloud computing to scale compute resources on demand. StochSS simulations can run on the users’ computer, or can make use of IaaS from the Amazon EC2 cloud. The *Cloud Computing* tool in StochSS is used to deploy and manage cloud resources and aims at making it easy also for a user inexperienced in cloud computing and systems administration to benefit from the possibility to transiently expand the computing capabilities of the system. To enable computations on the cloud, the user enters their AWS credentials and then launches one or more compute nodes. This tool enables the user to see the status of the compute nodes, select different types of nodes (for more advanced users), and to terminate the nodes. Once cloud compute nodes are running, simulations can be executed on these resources from the *Simulation Manager* by clicking the “Run on Cloud” button. Hence, the cloud computing capabilities of StochSS enable the expansion of the computing power available to the user at a click of a button, providing “Clusters On-Demand”.

### Deploying StochSS as Software as a Service to Enable Easy Collaboration and Resource Sharing

While many users will benefit from running StochSS as a local client with the ability to burst to the Amazon cloud to transiently increase the computing capabilities (i.e. a client-server setup), StochSS is also designed to work as multi-user Software as a Service (SaaS). Thus a research group can deploy StochSS as a collaborative environment on a shared server or in the cloud. Each individual user will then access StochSS through their web browser without the need to install any additional packages on their own computer, since both the GUI and all computations are run in the cloud. Individual users’ models can then be shared with the team by making them publicly viewable in StochSS. This option to deploy StochSS is a powerful feature for medium to large groups with multiple persons engaging in modeling projects. It can also facilitate collaboration across geographical boundaries, as it allows scientists to share a common modeling environment without the need to exchange models using other tools. A further use of deploying StochSS as a SaaS is for teaching, where the instructor can deploy and host a StochSS instance for all students to share, removing the need to configure and install all the software dependencies on the students’ own computers. MOLNs [22], another cloud framework in the StochSS suite of tools, targeted more at programmers and administrators, provides a Command Line Interface (CLI) to deploy and manage StochSS instances as SaaS in both public (EC2, Rackspace) and private (OpenStack, Eucalyptus) cloud environments. Executing a small set of intuitive commands is sufficient to set up a StochSS server to run as SaaS in a cloud IaaS.

### Example of Modeling and Simulation Using StochSS

A central design factor for StochSS has been the observation that modelers often want to start with a simple model in order to get experience with the system under study, and incrementally scale up the complexity of the model. This is reflected in the StochSS model editor, where the transition from a deterministic well-mixed model to a stochastic model, and then finally to a spatial stochastic model is made easy. To illustrate this, and to showcase the type of questions that can be addressed on the different modeling levels and by the different solvers and capabilities of StochSS, we formulated a simple, generic model of a gene regulatory network (GRN) with negative feedback regulation (self-repression). We chose this fundamental regulatory motif as our example since gene expression is part of many both small and large quantitative models, yet it is simple enough to clearly demonstrate how modeling is supported in StochSS.

We consider a single gene which, when its promoter *G*_*F*_ is unoccupied, is transcribed into mRNA. The mRNA is then translated into a transcription factor, protein *P*, which can in turn bind to the promoter, resulting in an occupied gene state *G*_*O*_. Once the promoter region is occupied by *P*, the transcription is suppressed. The model in its simplest form is written:

$$G_{F}\overset{\mu }{\to }G_{F}+mRNA$$ (1)

$$G_{F}+P\underset{k_{d}}{\overset{k_{a}}{⇋}}G_{O}$$ (2)

$$mRNA\overset{\kappa }{\to }mRNA+P$$ (3)

$$mRNA\overset{\gamma_{m}}{\to }\emptyset$$ (4)

$$P\overset{\gamma_{p}}{\to }\emptyset$$ (5)

For an initial parametrization of the model, we consulted Bionumbers [45], an online database of useful biological numbers that can greatly aid in quantitative modeling. For the size of the cell, we chose to model it after budding yeast, where the mean volume of diploid cells in exponential growth phase has been measured to 37 × 10^−15^ *L* [45, BNID 100430]. We use a median mRNA transcription rate in yeast of 0.12 mRNA molecules/min [45, BNID 106766] giving *μ* = 0.002*s*^−1^. The translation rate in budding yeast at 30°C is about 3-10 aa/s [45, BNID 107871]. Assuming a kinetic rate slowdown of about 2x per 10°C [45, BNID 100919] and using an average size of proteins of 467 aa [45, BNID 105224] we arrive at *κ* = 0.015*s*^−1^. For the degradation rates we use an average half-life of 40 min for proteins [45, BNID 104151] and 20 min for mRNA [45, BNID 106869], giving rate constants *γ*_*m*_ = 6 × 10^−4^ *s*^−1^ and $\gamma_{p}=\frac{\gamma_{m}}{2}$. For the binding rate of the protein to the promoter we use *k*_*a*_ = 10^7^ *Ms*^−1^ and for the dissociation *k*_*d*_ = 0.01*s*^−1^.

#### Simple well-mixed models highlight the main qualitative differences between modeling levels

In StochSS, this model is quickly constructed using the model editor in the WebUI, see Fig 2. We used ODE simulations and conducted a parametric sensitivity analysis around the base-line parameter set. Fig 3A shows a screenshot of the solution as seen in the StochSS UI. For these parameters the system quickly settles into a steady state, predicting a constant expression level of mRNA and transcription factor. A deterministic sensitivity analysis further suggests that the mean values are most sensitive to the degradation rates of protein and mRNA, see S1 Fig.

Previous models have shown how molecular noise can be a plausible mechanism to increase the robustness of genetic oscillators. For example, it was demonstrated in a seminal paper [5] that theoretically, near bifurcation points, intrinsic molecular noise can perturb the system from a stable fixed point and back onto a limit cycle. Thus, as the next step we asked in what way does intrinsic noise affect our model. We transition our model to a discrete stochastic model using the model conversion tool in StochSS. One additional parameter needs to be specified to switch modeling level: the system volume. Fig 3B shows a visualization of a single realization as seen by a user in the WebUI after issuing a simulation with SSA. Both the deterministic and stochastic models predict a reasonable absolute average copy number of transcription factor, i.e. on the order of hundreds to thousands [45, 109208]. However, only the stochastic model predicts bursty expression or apparent oscillatory behavior. To clearly illustrate this difference between the modeling levels we exported the simulation results from StochSS and plotted the ODE and SSA results in the same figure using Python plotting libraries (Fig 3C).

#### Scaling up the complexity to a spatial stochastic model

As the final step in our modeling process, we show how the model can be scaled up to a more detailed spatial stochastic model. Keeping the basic reaction network model the same, we now introduce a geometry representing a spherical yeast cell with a nucleus. The geometry consists of two concentric spheres modeling the cytoplasm and the nuclear compartments respectively. The gene is localized to a small region inside the nucleus, and transcription can only take place at that site. The mRNA diffuses in the nucleus and can eventually cross the nuclear membrane, exiting into the cytoplasm, where it can be translated. Fig 3D illustrates a computational mesh created using the external tool Gmsh [36] as imported into StochSS’s model editor. For the size of the nucleus subdomain (highlighted in red in the figure), we used 7% of the total cell volume [45, 104708]. For a baseline model, we chose typical values for the diffusion constants, D = 10^−12^ *m*^2^ *s*^−1^. Fig 3E shows a snapshot of a simulation trajectory as seen in the StochSS WebUI. For reference, we also exported the data and plotted the sum of protein in the entire domain with the different well-mixed results in Fig 3C. In this case, the spatial stochastic trajectory appears to result in qualitatively the same behavior as the well-mixed models. Indeed, the way we constructed this example, the spatial model we arrived at is structurally identical (however cell size and parameters are chosen based on another cell type) to the model used in [46] to study the effects of spatial locality and intrinsic noise on the Hes1 gene regulatory network. There it was found that this type of spatial stochastic model is, in contrast to both previous well-mixed stochastic models and spatial deterministic models, capable of capturing experimentally observed results well, both qualitatively and quantitatively.

## Discussion

StochSS is a software environment that makes it easy to create and simulate a model, starting simple and then scaling up with more detail. We anticipate this to be a common scenario in practice. However, there is a steep increase in computational cost associated with each model transition. For the deterministic ODE simulations, each simulation with identical starting conditions and parameters will result in the same simulation output, while individual realizations of stochastic models provide only one possible outcome of the model’s dynamical behavior. A rigorous analysis of a model then requires repeated independent sampling to form ensembles for statistical analysis. Even for the simplest well-mixed models this can be time consuming, and for spatial stochastic models it can become prohibitively expensive on a user’s laptop or even using high-end workstations. Table 1 illustrates the difference in computational cost of conducting the different simulations in the previous section.

**Table 1:** Illustration of simulation times and data output sizes for the different modeling levels supported by StochSS. There is a steep increase in computational cost as the model is refined.

Model	Wall clock time (s)	Size of Output (MB)
One ODE-solve	0.06	0.04
10^4^ well-mixed stochastic realizations	84	400
One spatial trajectory	197	7.6

It is clear from Table 1 that it is advantageous to use the simplest model that fulfills the need of a given modeling project, but this is often hard to assess in the beginning of a project. StochSS makes it possible to do bottom-up coarse-graining, starting from a detailed spatial stochastic model, generating time-series data and using the parameter estimation module of StochSS to fit parameters of a simpler, well-mixed model.

This paper has focused on describing the capabilities of StochSS, which centers on UI-based modeling and simulation and is targeted to users benefiting from a high level of abstraction. For most modeling projects, there will come a time where the full flexibility of a complete programming environment is needed. In this paper, this was illustrated by Fig 3C where we needed to combine data from multiple simulations and plot them in a way not supported by the UI. MOLNs [22] is another cloud framework we developed in the StochSS suite, targeted towards programmers and providing scalable, interactive parallel computing. In MOLNs, users work directly with the PyURDME and Gillespy APIs in IPython Notebooks and are able to easily generate ensembles and parameter sweeps. In future work, we plan for StochSS to directly access MOLNs’ functionality via the WebUI, making it possible to switch between WebUI and Notebooks for modeling, and to deploy and analyze large-scale parameter sweeps directly from the UI. To demonstrate the dual use of APIs and UIs, we provide a IPython/Jupyter Notebook in S2 Code that will run in MOLNs, showing how all the steps in the example in this paper can also be accomplished via programming.

## Availability and Future Directions

Stable releases of StochSS are available for download as binary packages at www.StochSS.org. Based on Docker containers, binary packages are available for OSX and Windows, and installation scripts are provided for Linux (Debian). The source code, including the latest development version, can be obtained from our GitHub repository, http://www.github.com/StochSS, where bugs and feature requests can also be reported.

Future directions include automation of the modeling process, interactive computing and integration with data. We are planning to add solvers for deterministic spatial simulations (partial differential equations) as well as microscopic and multiscale hybrid simulation methods for the spatial stochastic setting [47, 48]. We also plan to support more types of private, community and public cloud providers.

## Supporting Information

S1 Code Model files and simulation results for the example in the paper as a StochSS .zip archive. (ZIP)

S2 Code IPython/Jupyter notebook showing how all the models and experiments in S1 Code can be conducted using StochSS programming APIs. (IPYNB)

S1 Text Tutorial for using StochSS with the WebUI. (PDF)

S1 Fig Screenshot showing WebUI visualization of a deterministic sensitivity analysis. (PNG)
