# Supplementary material for: Stochastic Simulation Service: Bridging the Gap between the Computational Expert and the Biologist
Source: PLoS Comput Biol. 2016 Dec 8;12(12):e1005220. doi: 10.1371/journal.pcbi.1005220 (PMC5145134; doi:10.1371/journal.pcbi.1005220)
Supplement: S1 Text — (PDF) [file pcbi.1005220.s003.pdf]

UPPSALA
UNIVERSITET

StochSS: Stochastic Simulation Service

User Guide and Tutorial

StochSS
Stochastic Simulation Service

Contents

Contents **2**

1 Installation **4**
- 1.1 Try StochSS 4
- 1.2 Install StochSS 4

2 Basic Introduction **5**
- 2.1 Creating Administrator and Standard User Accounts 5
- 2.2 Importing a Model 6
- 2.3 Creating a New Model 7
- 2.4 Running a Simulation and Visualizing results 8
- 2.5 Backup and Transfer your Data 9

3 Sensitivity Analysis **11**
- 3.1 Example: Michaelis-Menten 11

4 StochOptim: Parameter Estimation **12**
- 4.1 Example: Estimate parameters of a birth-death model 12

5 Spatial Stochastic Simulations **14**
- 5.1 Example: Annihilation of two species in a cylinder 14

6 Cloud Computing **16**
- 6.1 Credentials 16
- 6.2 Job Reproduction 17
- 6.3 Cost Analysis 18

7 Parameter Sweeps **20**

8 Visualization **22**
- 8.1 Surface Renderings with Domain Clipping 22
- 8.2 Colorized Wireframe Meshes 23
- 8.3 Volume Rendering 23

8.4 Visualization in Interactive Jupyter Notebook 23

9 Import a custom mesh 25
9.1 Mesh library 25
9.2 Using DOLFIN Built-In Geometry with Subdomains Outside of Mesh Library 25
9.3 Advanced mesh generation 27
9.4 References 28

Bibliography 29

1 Installation

1.1 Try StochSS

It is possible to try StochSS online at try.stochss.org by signing up with your e-mail address. Nothing has to be installed locally, and all modeling capabilities of StochSS are available. It is *not* possible to take advantage of advanced cloud capabilities (see Section 6) through the try.stochss.org-server, so for the user with the need to run large-scale computations, we recommend to consider a local install after trying out StochSS online.

1.2 Install StochSS

StochSS supports Mac OS X, Linux, and Windows through the Docker platform. To install StochSS, you will need to install Docker on your computer, and then use the appropriate StochSS launch application to initialize and run your StochSS Docker container. Docker is free software that automates the deployment of Linux applications inside software containers. To download Docker, visit https://www.docker.com/.

For instructions on how to install the latest version of StochSS for your platform, please see the StochSS website http://www.stochss.org/. The source code, together with installation instructions, is also available on GitHub: https://github.com/StochSS/stochss/tree/master/stochss-launcher.

2

Basic Introduction

This tutorial will guide you through the basic features of StochSS. You will become familiar with the **Model Editor** and the **Simulation Manager**. You will learn how to create your own model, which can be population or concentration-based, and how to simulate it using either an ordinary differential equation (ODE) solver or the stochastic simulation algorithm (SSA).

2.1 Creating Administrator and Standard User Accounts

At the end of a successful installation process, your default browser will launch and you will be asked to create an admin account as in Figure 2.1 (there is only one admin account for the entire system). Once the admin account is created you will be forwarded to a regular login page where you can log in to StochSS.

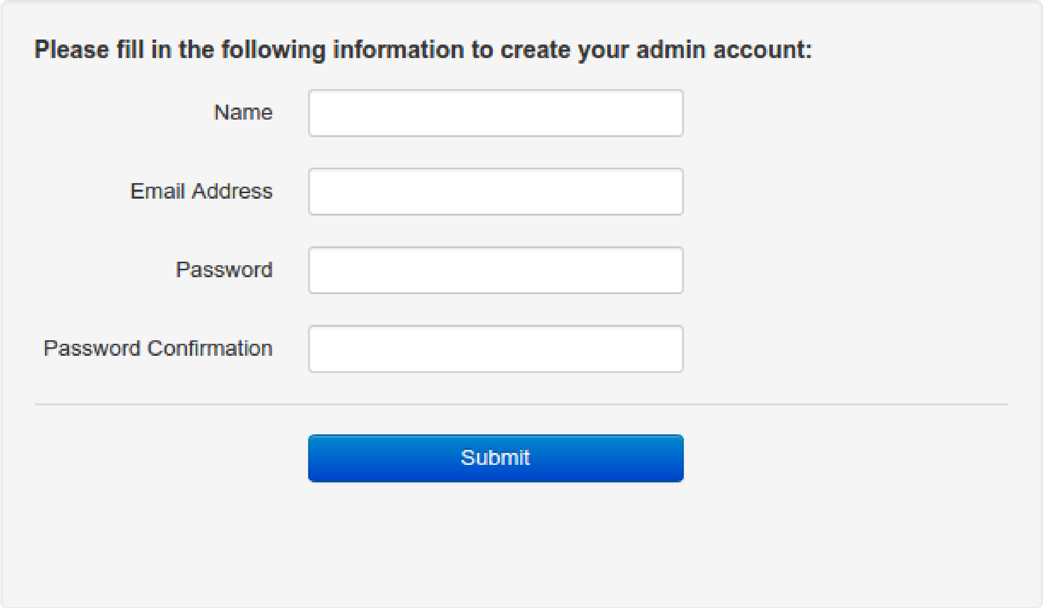

Figure 2.1: Administrator login page

Users can click **Create Account** to request an account. The account will not be accessible until the admin approves it in the **Admin Panel** (the admin can also delete active users as well as reset their passwords there).

2.2 Importing a Model

The **Model Editor** will let you create new or modify existing well-mixed stochastic biochemical models as well as deterministic models based on ODEs. The best way to get started with the model editor is to import an example model and look at the different sections.

Importing an existing model

Option 1: StochSS Public Library

1. Navigate to the **Model Editor** page.
2. Click **Import from Public Library** in the right-hand toolbar.
3. Select a model from the Public Library and click **Copy Model to Library**.

Option 2: Stochkit2 XML

1. Navigate to the **Model Editor** page.
2. Click **Import from .XML** in the right-hand toolbar.
3. Select an XML file. A collection of example models can be found in the *examples* directory within the StochSS install folder.
4. Click **Import**.

Option 3: SBML

1. Navigate to the **Model Editor** page.
2. Click **SBML** in the right-hand toolbar.
3. Select an SBML file and click submit.

Note that due to limitations with the SBML standard, StochSS only supports non-spatial models in this format.

After importing the model, StochSS should display the imported model in the model editor. Look through the page to see how the different **Species**, **Parameters**, and **Reactions** are defined.

By clicking **Export to .zip**, **Export to Public Library**, or **Export to SBML**, the model can be shared across computers or shared amongst users of the same StochSS.

2.3 Creating a New Model

An example population model is defined by the following two reactions:

$$\begin{aligned} S0 + S0 &\xrightarrow{k1} S1 \\ S1 &\xrightarrow{k0} \emptyset. \end{aligned} \tag{2.1}$$

To create this model:

1. Navigate to the **Model Editor**.
2. Click **Add Model** and select *Population, Well-mixed.*
3. Rename the model to *example.*
4. Click **Create Species** twice to create two species.
5. By default the species are named $S0$ and $S1$. Set the initial condition for $S0$ to 1000 and the initial condition for $S1$ to 0.
6. Similarly to above, click **Add Parameter** twice to add two parameters.
7. By default they will be named $k0$ and $k1$. Set $k0$ to 0.0001 and $k1$ to 0.05.
8. Click **Add Reaction** to add two reactions. Select the reactants, products, rates and reaction types corresponding to (2.1). Compare to Figures 2.2 and 2.3 to verify the settings.

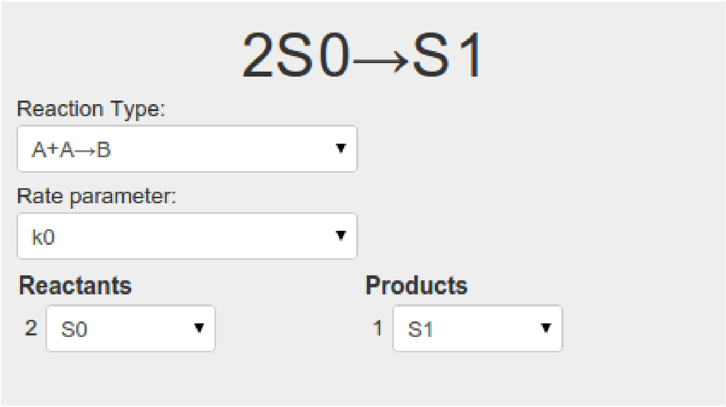

Figure 2.2: Dimerization reaction

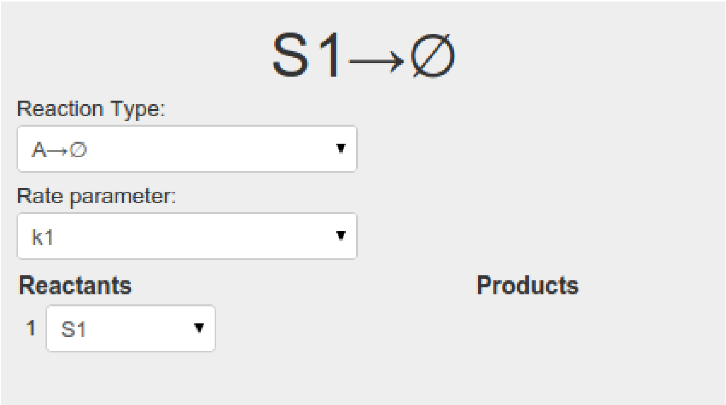

Figure 2.3: Decay reaction

2.4 Running a Simulation and Visualizing results

For this section, create or import a model using the directions above.

1. Navigate to the **Simulation Manager** page.
2. Select the model you wish to simulate and click **Next**.
3. Setup your simulation parameters: name, time, data storage frequency, realizations and solver type.
   a) If you are simulating a population-based model you can choose between the deterministic and the stochastic solvers.
   b) Concentration-based models can only be simulated using the deterministic solver.
4. Click **Run Locally**. You will be automatically forwarded to the **Job Status** page.
5. Click **View** to open the **Job summary** page, where you can visualize the simulation's trajectories.
6. Click **Access Local Data** to download a raw copy of the data. This can be used to share data between StochSS installations or perform manual data analysis.

Converting a concentration model to population

Create a concentration model or use the directions above to import one. Both Lotka-Volterra examples are concentration based and are available both as XML files and Public Library models.

1. Select the newly minted concentration model.
2. Click **Convert to Population** on the right-hand toolbar to start the conversion process. The model conversion page will open.
3. To convert a concentration model to a population, a system volume must be specified.

4. Given a volume, StochSS converts initial conditions through

$$\text{initial\_condition\_population} = \text{initial\_condition\_concentration} \times \text{volume} \tag{2.2}$$

   and attempts to convert the reaction rates. It is not always possible to convert reaction rates automatically. During the conversion process StochSS lists all the reactions, and notifies the user of which reaction rates were successfully converted and which were not. If automatic conversion fails the conversion still proceeds, but the user now has to correct the reaction rates that were not automatically converted.

5. Click **Finish conversion** at the bottom left of the page to create a population-based model. This newly created population model can be simulated using both deterministic and stochastic solvers.

6. Click **Cancel conversion** to cancel the conversion.

The conversion process operates correctly only if the model to be converted is entirely based on the mass action kinetics allowed in Gillespie Stochastic Simulation Algorithm [1]. If the model to be converted is not entirely based on mass action kinetics, the conversion tool only converts what it can.

2.5 Backup and Transfer your Data

You can backup or share your saved models and simulations with the StochSS ZIP format. There are three ways to create StochSS ZIP files:

- Navigate to the **Backup** page in the left-hand toolbar and click **Export**. This exports a ZIP containing all models and simulation results for the current user. There is an option to export all data for all users if this page is accessed with the admin account.
- Select a model on the **Model Editor** page and click **Export to .zip**.
- Click **View** on the **Job Status** page, and then click **Access local data**.

These ZIP files can all be imported into StochSS on the **Backup** page. To import the contents of a ZIP file into StochSS:

1. Navigate to the **Backup** page.
2. Click **Import**.
3. Select the ZIP file to upload. The file should automatically begin uploading, and then appear in a table of ZIP archives below.
4. Select the ZIP file in the table.
5. Define the behavior of the import by either limiting what files get imported or specifying how overlapping names are handled.
6. Click **Import** at the bottom of the page.

Exporting data from an old version of StochSS (1.2 or previous)

To create a backup archive from an older version of StochSS, execute the following command from a terminal window in the directory of your new StochSS installation:

```
./exportserver.py path_to_your_old_StochSS_installation
```

You can import the backup archive you created as described above.

3

Sensitivity Analysis

StochSS implements forward sensitivity analysis based on the CVODES ODE solver [2].

For instance, if we consider the population of P and the parameter $Vmax$ in the Michaelis-Menten model in the **Public Library**. The sensitivity of the rate of the increase in population P to the parameter $Vmax$ is defined as the incremental rate of change in P due to incremental changes in $Vmax$:

$$\frac{\partial P(t)}{\partial V_{max}}. \tag{3.1}$$

These are the sensitivities that StochSS produces. There is no rescaling to make these sensitivities unitless.

3.1 Example: Michaelis-Menten

In this example we will study the Michaelis-Menten model from the StochSS Public Library. Details on the model can be found in [9].

1. Import the model from the public library. By default, it will be called *michaelis_menten*.
2. Navigate to the **Simulation Manager**, select the model, and click **Next**.
3. Under **Simulation type**, select *Deterministic + Sensitivity*.
4. You can now select the parameters for which you wish to compute sensitivities. For instance you could select Km, $k3$, mu, and $Vmax$, to perform sensitivity analysis with only these parameters.
5. Click **Run Locally**.
6. Navigate to the **Job Summary** page to analyze the output. You can plot species trajectories as well as sensitivities.

4

StochOptim: Parameter Estimation

StochSS implements parameter estimation for stochastic well-mixed biochemical systems, StochOptim, via the Monte Carlo expectation-maximization with Modified Cross-Entropy method (MCEM^2) [5]. MCEM^2 computes maximum likelihood parameter estimates (MLEs) and associated uncertainties in three consecutive phases: cross-entropy, Monte Carlo expectation-maximization (MCEM), and uncertainty quantification [5].

4.1 Example: Estimate parameters of a birth-death model

We consider the Birth-Death model that can be imported from the StochSS Public Library (see Section 2 for instructions on how to import models in StochSS). After the model has been imported:

1. Select **Parameter estimation** from the menu on the left of the screen.
2. Select the *birthdeath* model and click **Next**. This will open the **Simulation page**.
3. Example files (StochOptim input data) for the initial conditions and the trajectories are provided in the *examples* folder (included in your StochSS package) and are named *birthDeathInitial.txt* and *birthDeathTrajectories.txt*, respectively.
4. Upload and select these example files.
5. Configure the checkboxes to only perform parameter estimation on $k1$. For the example model, the value of $k2$ is already optimized.
6. Click **Run Locally** to start the parameter estimation.
7. Select **Job Status** from the menu on the left of the screen to monitor the status of your submitted job. For the example trajectory data, $k1 = 1.0$ and $k2 = 0.03$ are the correct parameter values so the simulation should converge to somewhere close to these.
8. Click **View Progress** to access the **Job Summary** page and to view more details about status of your job. Parameter estimation calculations using MCEM^2 are typically time consuming computations. This simple example takes a little less than 20 minutes on a desktop Intel i7 computer. A more realistic job will take much longer to run.
9. When the job has finished, you can generate a new model using the final estimates of the MCEM^2 calculation by scrolling to the bottom of the **Job Summary** page and click **Create Model from Current Estimates**.

10. If the job doesn't complete in a reasonable amount of time the job can be stopped manually on the **Job Status** page and the parameters can be extracted by clicking **Create Model from Current Estimates**.

StochOptim input file format

The StochOptim input data format consists of two tab-separated text files. The first file represents the initial conditions of the system, and the second file represents trajectories of it. Each file has three base columns. *Time*, *Rep*, and *Weight*. Additional columns are added for every species in the model that parameter estimation is to be run on.

- The *Time* column contains the time values of the various trajectories (and should be set to zero for the initial conditions file).
- The *Rep* column is used to include multiple trajectories in one file for fitting. Basically each trajectory should have a different *Rep* number.
- The *Weight* column is currently unused and should be set to 1.
- The rest of the columns should be named after the species (case-sensitive) in the model the data will be fit against, and the columns themselves should contain integers representing the population counts at the various time points.

An example of this can be seen by comparing the files *birthDeathTrajectories.txt* and *birthDeathTrajectoriesMulti.txt* in the *examples* folder included in the StochSS package.

5

Spatial Stochastic Simulations

The spatial stochastic simulation capabilities in StochSS are based on PyURDME [8]. PyURDME is a general software framework for modeling and simulation of stochastic reaction-diffusion processes on unstructured, tetrahedral (3D) and triangular (2D) meshes. The current core simulation algorithm is based on the mesoscopic reaction-diffusion master equation (RDME) model. The default solver is an efficient implementation of the next subvolume method (NSM) [7].

5.1 Example: Annihilation of two species in a cylinder

We will build a simple annihilation model based on an cylinder geometry. At each end of the cylinder, different chemicals will be produced. When they diffuse and meet at the center, they will annihilate each other.

1. Navigate to the main **Model editor**.
2. Add a new model. Select *Population, spatial* in the dropdown menu.
3. Click **Mesh** and select *Cylinder*. The cylindrical mesh is divided into three subdomains which can be visualized with the controls below the wireframe view.
4. Add two species, A and B, both with diffusion constant 1.
5. Click **Initial Condition**, select *scatter*, and add 500 molecules of species A in subdomain 1 and 500 molecules of species B in subdomain 3.
6. Add two parameters, $k0$ and $k1$, and set their values to 1 and 100, respectively.
7. Add three reactions:

$$\begin{aligned} \text{R1}: &\quad \emptyset \xrightarrow{k_1} A \\ \text{R2}: &\quad \emptyset \xrightarrow{k_1} B \\ \text{R3}: &\quad A + B \xrightarrow{k_0} \emptyset \end{aligned}$$

8. Reaction $R1$ should be restricted to subdomain 1 and reaction $R2$ to subdomain 3. Reaction $R3$ should be allowed throughout the whole domain.
9. The model is now complete and ready to be simulated.

10. Navigate to the **Simulation manager** page.
11. Select the spatial model you just created and click **Next**.
12. Setup the simulation parameters: name, time, data storage frequency, and number of realizations.
13. You can specify a random seed for the random number generator under **Advanced Settings**.
14. Click **Run locally**.
15. In a few seconds you will be directed to the **Job Status** page where you can check the status of your simulation.
16. Once your simulation is complete, click **View results** to open the **Job summary** page, where you can visualize the diffusion of the two species over time within the cylindrical container and download the output files of the simulation.

6

Cloud Computing

6.1 Credentials

StochSS provides the options to run jobs using the Amazon cloud infrastructures. In order to use Amazon Elastic Computing Cloud (EC2), Simple Storage Service (S3) and DynamoDB database, which are all required for running jobs in the cloud, you need an Amazon Web Services (AWS) account and a credential pair (consisting of a secret key and an access key) in hand.

More information regarding how to create an AWS account and get credentials can be found here: `http://aws.amazon.com`

Setting Credentials in StochSS

You must set your credentials in StochSS manually (Figure 6.1). Once StochSS validates these, you will be able to launch compute nodes and run jobs in the Amazon cloud.

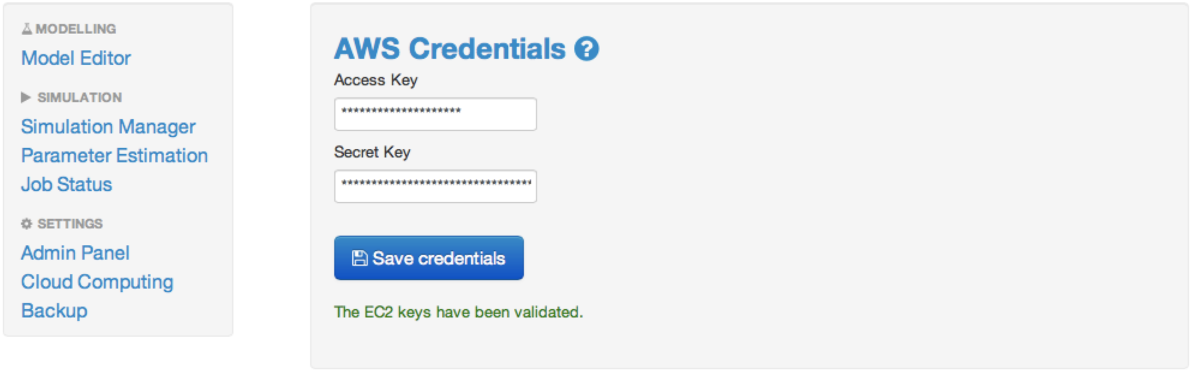

Figure 6.1: Cloud Computing page - Credentials section

1. Navigate to the main **Cloud Computing** page.
2. Copy your access key to the **Access Key** text box.
3. Copy your secret key to the **Secret Key** text box.

4. Click **Save credentials** to validate and save your credentials.

Launching and Shutting Down Nodes

Once valid credentials are entered, clicking **Launch nodes** (Figure 6.2) by default will launch one c3.large Amazon instance. This first node is designated the **head node**. Any other nodes launched are called **worker nodes**. There can be zero or more worker nodes, and they are c3.larges instance types by default. For information on Amazon instance types, look here: `http://aws.amazon.com/ec2/instance-types/`. The head node must be a c3.large or c3.xlarge. The worker nodes can be any combination of t1.micro, m1.small, m3.medium, m3.large, c3.large, and c3.xlarge nodes (chosen in the **Advanced settings** menu).

Only one head node is needed to run jobs in the cloud. It is possible to access cloud data even when no head nodes are launched. Compute resources and storage resources are billed separately by Amazon. More details can be found at: `http://aws.amazon.com/ec2/pricing/`.

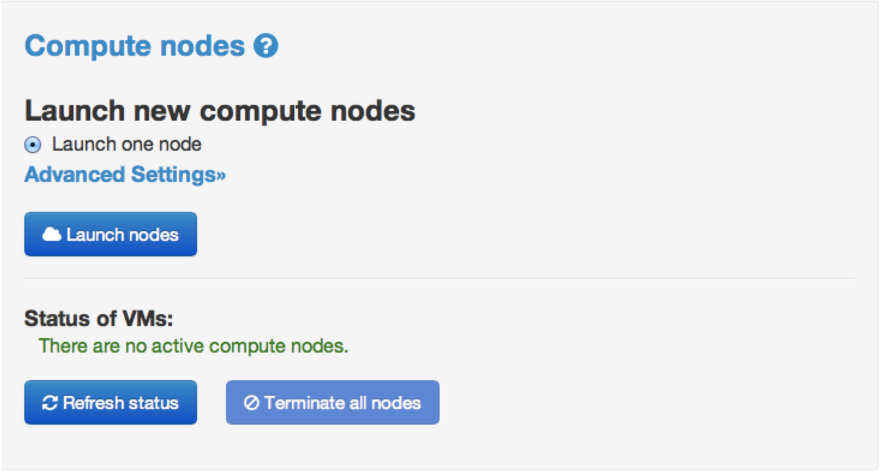

Figure 6.2: Cloud Computing page - Compute nodes default setting section

Launching a node takes time. The **Refresh status** button can be used to check the launch progress. The **Terminate all nodes** button terminates all the nodes that StochSS started.

6.2 Job Reproduction

StochSS provides the flexibility to store simulation output in the cloud or delete it and regenerate it later. If simulations are fast but produce large amounts of data, reproducing data only when it is needed can save money.

An Example on Job Reproduction

Reproducing a cloud job is simple:

1. Launch a compute node.

2. Run a well-mixed or spatial model (everything except parameter estimation jobs can be reproduced).

3. Navigate to the **Job Status** page.

4. Click **view** beside the job you would like to reproduce.

5. Click **Delete Output** to delete output in the cloud. No reproduction action is available until you delete the output.

6. Once the output is deleted, the option to reproduce the job will be appear as shown in Figure 6.3.

7. Choose a node type for reproduction. If there is no such instance type running, a warning will show up to guide you to the **Cloud Computing** page to launch one.

8. Click **Reproduce Results** to submit the reproduction request. This will automatically redirect to the **Job Status** page where the new job's status can be monitored.

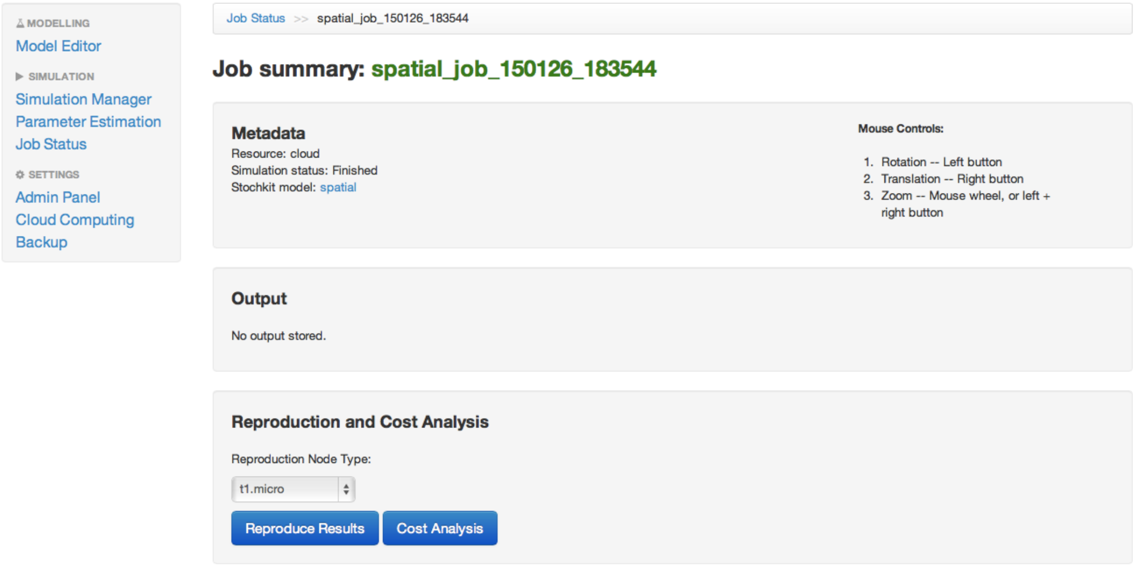

Figure 6.3: Job summary page - reproduction available

6.3 Cost Analysis

Because different instance types cost different amounts of money, it is not obvious which nodes are the cheapest for any given job type. StochSS allows manual measurement of job cost with the cost-analysis tool.

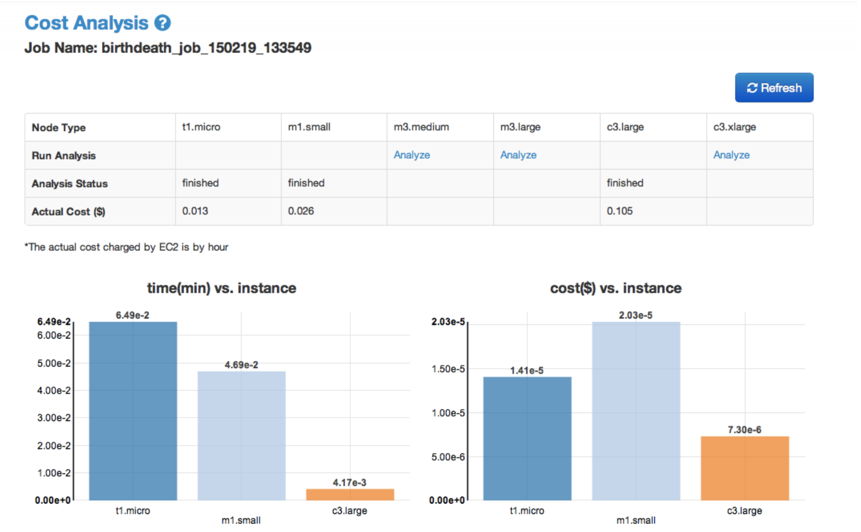

Figure 6.4: Cost Analysis page

An Example on Job Reproduction

1. Run a well-mixed or spatial model.
2. Navigate to the **Job Status** page.
3. Click **view** beside the job you would like to analyze.
4. Click **Cost Analysis** in the **Reproduction and Cost Analysis** section.
5. By default, cost analysis should be available for whatever instance type the job was run on.
6. Click **Analyze** with any other node type you would like to run and analyze the job on. At least one node of this instance type must already be running.
7. The run times and costs of simulating the jobs are plotted on the screen as in Figure 6.4.

7

Parameter Sweeps

It is often of interest to explore the parameter space of a model. We may want to find what the value of some parameters should be, or maybe we want to find regions in parameter space where the model exhibits interesting behavior.

StochSS supports sweeps over one or two parameters. To set up a parameter sweep, simply click **Parameter Sweep**. Select the model that you want to analyze, the parameters you want to analyze, the upper and lower bound of the parameters, and the number of steps in each parameter.

First import the *lotkavolterra_concentration_oscil* model from the *Public Library*. Select this model in the parameter sweep main window, and run a two-parameter sweep over $k1$ and $k2$. Click **Run Local**, and wait for the computation to finish. Once it has finished, the output can be visualized on the **Job Summary** page, see Figure 7.1. You have to select a *mapper* and a *reducer*, and can view the average concentrations, the max- and min-values, as well as the variance. As an example, if the *mapper* is 'final time', and the reducer is 'average', then that means that you will plot the average of the populations at the final time point. Another example is that the mapper is 'average' and the 'reducer' is 'max'. That means that we first take the average of each trajectory, and then the maximum of each average. The final output is then visualized in a graph or heatmap.

Finally, if you need to perform an in-depth analysis of the data, there is the option of exporting the parameter sweep to a Jupyter Notebook. On the **Job Summary** page, click **Analyze using interactive Jupyter Notebook**. This launches a Jupyter Notebook, with a template for analyzing the data. We recommend users unfamiliar with Jupyter to visit http://jupyter.org for full documentation and tutorials. In short, it is an environment for interactive computing, and users can analyze and plot their data with Python scripts. For example, given a two-parameter sweep, we may ask ourselves which parameter values give a specific output value. While it is possible to estimate that by simply looking at the heatmap, that is likely unsatisfactory in a real modeling project. With the full scripting capabilities of Python, the user could automate and make this process rigorous by modifying the Notebook correspondingly.

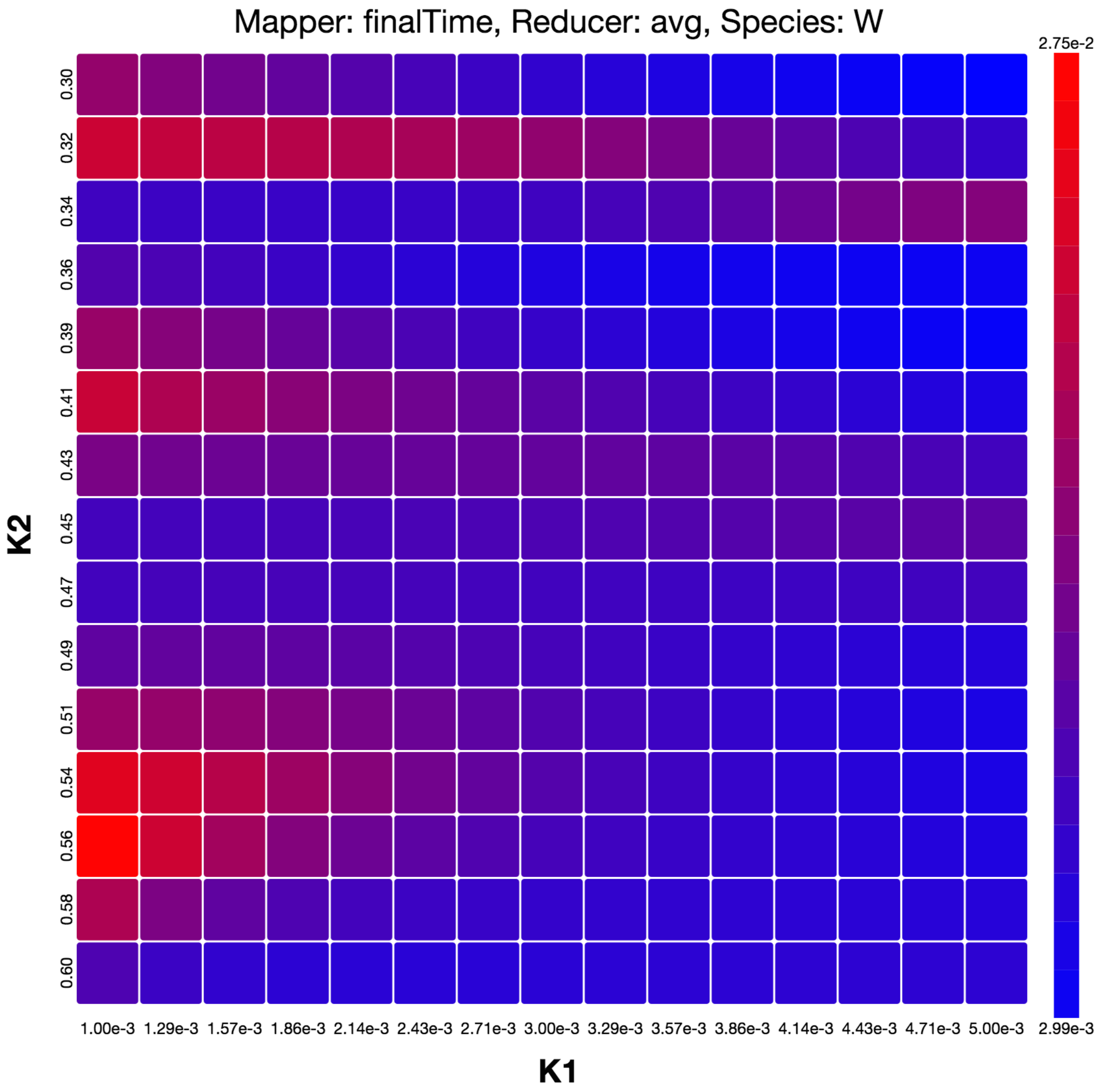

Figure 7.1: A two-parameter sweep is visualized as a heat map. We have performed a sweep over the two parameters $k1$ and $k2$ of the *lotkavolterra_concentration_oscil* model. The *mapper* performs the initial analysis on the output—in this case simply taking the value of each solution at the final time point. The *reducer* performs some analysis on the output of the mapper—in this case taking the average of the final time points. Note that this model is deterministic, and the average simply is the value of the ODE solution at the final time point.

8

Visualization

Full three-dimensional spatial stochastic simulations can be difficult to analyze. To simplify the process, StochSS has built in three different methods for visualization of spatial models.

8.1 Surface Renderings with Domain Clipping

By default, spatial stochastic simulations are rendered as shown in Figure 8.1. These are surface renderings, but the simulations are volumetric. To see inside the volume, StochSS allows slicing the mesh in half along one axis with a plane and only rendering one of the resultant halves. This is shown in Figure 8.2

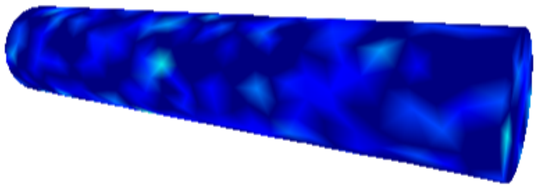

Figure 8.1: Surface rendering of cylindrical domain. The actual stochastic simulation is run on the dual of the shown mesh, so the color at each node corresponds to the concentration in the corresponding voxel of the dual mesh. Colors are interpolated linearly between nodes. The color scale is not shown for brevity.

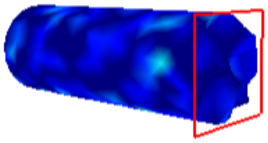

Figure 8.2: Surface rendering of cylindrical domain clipping along the X dimension. It is now possible to see concentrations of voxels inside the cylinder.

8.2 Colorized Wireframe Meshes

The second rendering type is similar to the first, but instead of rendering surfaces only edges are rendered (see Figure 8.3). The colorization is the same, it is just ideally easier to see inside the mesh. Similarly to the surface rendering the wireframe renderings can be clipped to get a clearer view of what is happening inside the volume. See Figure 8.4 for a demonstration of clipping a mesh in the Y dimension.

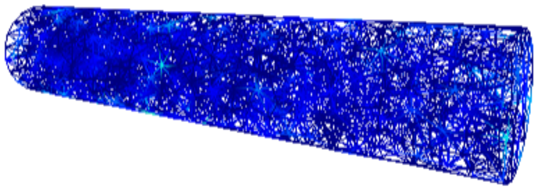

Figure 8.3: Wireframe rendering of cylindrical domain. The colors are handled similarly to in Figure 8.1.

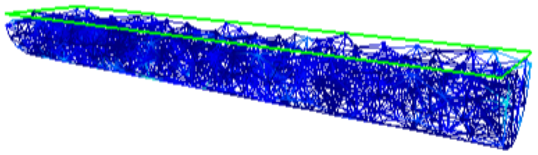

Figure 8.4: Wireframe rendering of cylindrical domain clipped in the Y direction.

8.3 Volume Rendering

The final rendering StochSS offers is a volume rendering (Figure 8.5). It uses a basic ray-tracing implementation following the one in [10].

8.4 Visualization in Interactive Jupyter Notebook

In many projects the need to perform custom postprocessing and visualization will eventually arise. To facilitate this process, StochSS offers the possibility to export a Jupyter Notebook template, which reads the output and plots the average populations of the species. The user can then customize the Notebook to perform the required postprocessing and plotting.

To access this function, simply click **Analyze using Interactive Notebook** on the **Job Summary** page. Figure 8.6 shows the default template.

For documentation and tutorials on Jupyter Notebooks, see http://jupyter.org.

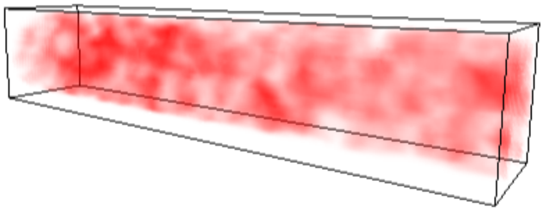

Figure 8.5: This is the volume rendering of the same data shown above. The idea behind volume rendering is to color darkly areas with high concentrations and leave volumes with low concentrations transparent. The transparency makes it possible to see inside a volume rendering, and so the slicing as shown in the surface and wireframe renderings is not used.

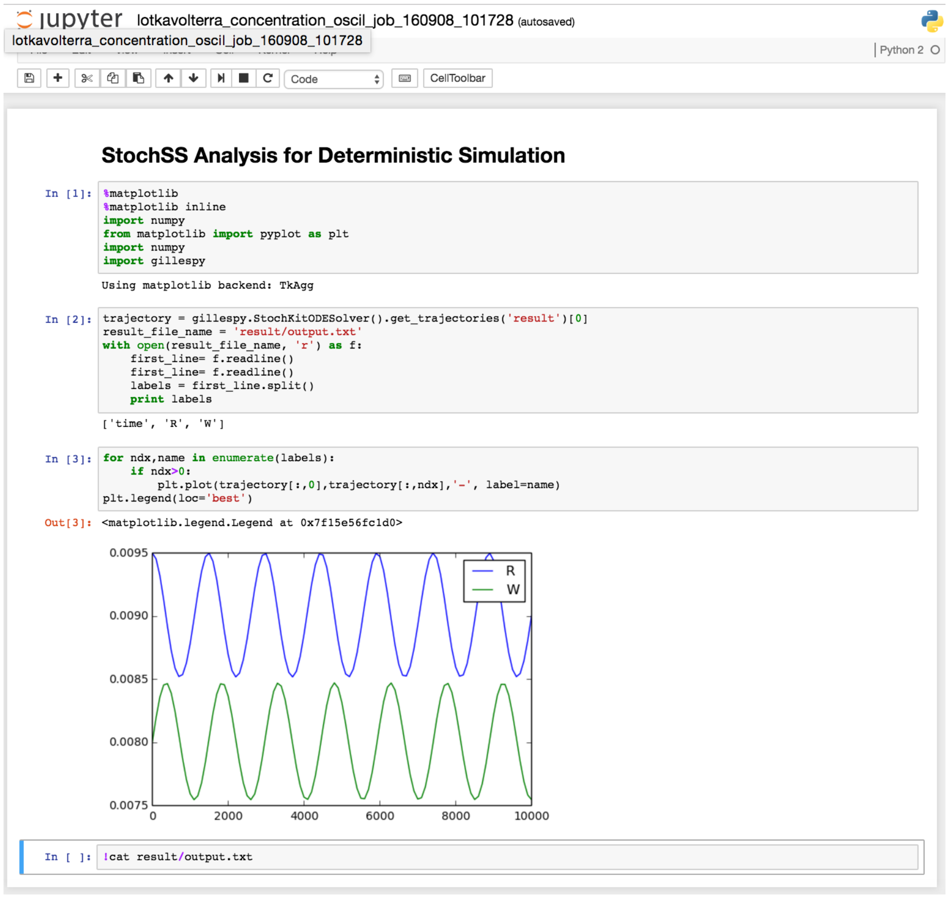

Figure 8.6: Default Notebook for custom postprocessing.

9

Import a custom mesh

When setting up a spatial problem in StochSS information about the geometry needs to be provided through two files: a file containing the mesh information in DOLFIN XML format and a text file containing information about subdomains. The file with subdomain information is optional in general (if it is not provided the entire mesh will belong to one subdomain). Below is a tutorial on how to provide that information in a variety of cases.

9.1 Mesh library

The first and simplest scenario is using one of the default built-in meshes provided by StochSS in the Mesh Library. These include simple example mesh and subdomain combinations that are common in biological modeling. For example there is a unit sphere with a membrane (i.e. the volume is one subdomain and the surface is another), a unit cube witha membrane, a cylinder with the two ends being different subdomains among others. If for a given problem these are not sufficient then mesh and subdomain information can be provided by attaching files. One simple case where creating and attaching a file may be necessary could involve using the DOLFIN built in meshes but with different subdomains than provided in the Mesh Library. This can be done as follows.

9.2 Using DOLFIN Built-In Geometry with Subdomains Outside of Mesh Library

For example consider a sphere with three different subdomains: the top half of the membrane, the bottom half of the membrane and the volume. This can be done using PyURDME as follows (PyURDME and FEniCS are software packages that are automatically installed during the StochSS installation and DOLFIN is a library within FEniCS).

In an IPython Notebook, import the appropriate libraries:

In [1]:
```
import os,pyurdme,dolfin
```

Next classes are constructed to define the desired subdomains:

In [2]:
```
class top_half_membrane(dolfin.SubDomain):
        def inside(self,x,on_boundary):
        return on_boundary and x[2]>=0

class bottom_half_membrane(dolfin.SubDomain):
        def inside(self,x,on_boundary):
        return on_boundary and x[2]<=0
```

Then a pyurdme model is created with the desired geometry and marked subdomains:

In [3]:
```
class sphere_top_bot(pyurdme.URDMEModel):
        def __init__(self,model_name="polarization"):
                pyurdme.URDMEModel.__init__(self,model_name)

                #DefineGeometry
                sphere=dolfin.Sphere(dolfin.Point(0.0,0.0,0.0),1.0)
                self.mesh=pyurdme.URDMEMesh(mesh=dolfin.Mesh(sphere,10))

                cell_function=dolfin.CellFunction("size_t",self.mesh)
                cell_function.set_all(1)

                facet_function=dolfin.FacetFunction("size_t",self.mesh)
                facet_function.set_all(0)

                top_membrane=top_half_membrane()
                top_membrane.mark(facet_function,2)

                bottom_membrane=bottom_half_membrane()
                bottom_membrane.mark(facet_function,3)

                self.add_subdomain(cell_function)
                self.add_subdomain(facet_function)

                #Define initial populations
                self.set_initial_condition_scatter({},[1])
```

In [4]:
```
model = sphere_top_bot()
```

The subdmain infomation can then be extracted from the model as follows:

In [5]:
```
sd = model.get_subdomain_vector()
```

And finally two files are created to store the mesh and subdomain information respectively in the the correct format:

In [6]:
```
 This is a dolfin xml file with mesh information
dolfin.File("sphere_top_bot_mesh.xml") << model.mesh
```

In [7]:
```
 This is the file to capture information about subdomains
with open("sphere_top_bot_subdomains.txt",'w') as fd:
        for ndx,val in enumerate(sd):
                fd.write("{0},{1}\n".format(ndx,val))
```

Now that these two files have been created they can be attached in the Mesh tab of the model editor in StochSS and the rest of the problem can be defined from there. Again it is important to note that if a problem does not involve subdomains then only the mesh XML file is necessary and the entire mesh will be given the same domain! One last situation that could arise in defining spatial information is that a mesh has to be created in external software then converted to the correct format.

9.3 Advanced mesh generation

For many real world applications the geometry involved will be more complicated than the built?in meshes provided by DOFLIN (and by extension StochSS). In these situations a mesh will have to be created in an external program such as Gmsh then converted into the DOLFIN XML format. Once the mesh has been created in an external program the conversion to DOLFIN XML format can easily be done by the built in script dolfin?convert [1]. The following command line code demonstrates how to convert from the Gmsh format (suffix .msh or .gmsh) to DOLFIN XML format.

The following needs to be run from the command line in the Terminal:

```
dolfin-convert mesh.msh mesh.xml
```

File formats that are currently supported by the dolfin-convert script can be found in the table below:

Suffix	File Format
.xml	DOLFIN XML format
.ele / .node	Triangle file format
.mesh	Medit format, generated by TetGen with option ?g
.msh / .gmsh	Gmsh version 2.0 format
.grid	Diffpack tetrahedral grid format
.inp	Diffpack tetrahedral grid format
.e / .exo	Sandia Exodus II file format
.ncdf	ncdump'ed Exodus II file format
.vrt / .cell	StarCD tetrahedral grid format

The XML file that is created from the dolfin?convert command can then be used directly in StochSS. If subdomains are required then a text file can be created using code similar to that above. Consider the situation where the XML file created was called "coli.xml" then the code to create a subdomain file consisting of the outer membrane would look as follows:

In [8]:
```
import os,pyurdme,dolfin
```

In [9]:
```
class Membrane(dolfin.SubDomain):
        def inside(self,x,on_boundary):
        return on_boundary
```

In [10]:
```
class ecoli(pyurdme.URDMEModel):
        def __init__(self,model_name="polarization"):
                pyurdme.URDMEModel.__init__(self,model_name)

                #DefineGeometry
                self.mesh=pyurdme.URDMEMesh(mesh=dolfin.Mesh('coli.xml'))

                cell_function=dolfin.CellFunction("size_t",self.mesh)
                cell_function.set_all(1)

                facet_function=dolfin.FacetFunction("size_t",self.mesh)
                facet_function.set_all(0)

                membrane=Membrane()
                membrane.mark(facet_function,2)

                self.add_subdomain(cell_function)
                self.add_subdomain(facet_function)

                #Define initial populations
                self.set_initial_condition_scatter({},[1])
```

In [11]:
```
model=ecoli()
```

In [12]:
```
sd=model.get_subdomain_vector()
```

In [13]:
```
This is the file to capture information about subdomains
with open("coli_subdomains.txt",'w') as fd:
        for ndx,val in enumerate(sd):
                fd.write("{0},{1}\n".format(ndx,val))
```

Now all of the necessary files have been created. The "coli.xml" file that was created using a dolfin?convert command and the "coli_subdomains.txt" just created can be uploaded directly to StochSS and used accordingly.

9.4 References

1. A. Logg, K.?A. Mardal, G. N. Wells et al. Automated Solution of Partial Differential Equations by the Finite Element Method: The FEniCS Book. Springer 2012.

Bibliography

[1] D.T. Gillespie. *Exact stochastic simulation of coupled chemical reactions.* J. Phys. Chem., 81(25), 2340-2361 (1977)

[2] A. C. Hindmarsh et al., *SUNDIALS: Suite of nonlinear and differential/algebraic equation solvers.* ACM Trans. Math. Softw., 31(3), 363-396 (2005)

[3] W. A. Link and P. F. Doherty Jr., *Scaling in sensitivity analysis.* Ecology, 83(12), 3299-3305 (2002)

[4] M. C. Hill, *Methods and guidelines for effective model calibration.* U.S. Geol. Surv. Water Resour. Invest. Rep. 98-4005 (1998)

[5] B.J. Daigle et al. *Accelerated maximum likelihood parameter estimation for stochastic biochemical systems.* BMC Bioinformatics, 13, 68 (2012)

[6] B.S. Caffo et al. *Ascent-based Monte Carlo expectation-maximization.* J. Royal Statistical Society Series B, 67(2), 235-251 (2005)

[7] J. Elf and M. Ehrenberg, *Spontaneous separation of bi-stable biochemical systems into spatial domains of opposite phases.* IEEE Systems Biology, 1, 230-6 (2004)

[8] B. Drawert, S. Engblom and A. Hellander, *URDME: A modular framework for stochastic simulation of reaction-transport processes in complex geometries.* BMC Systems Biology, 6(76) (2012)

[9] Michaelis-Menten kinetics. `http://en.wikipedia.org/wiki/Michaelis-Menten_kinetics`

[10] Congote, John and Segura, Alvaro and Kabongo, Luis and Moreno, Aitor and Posada, Jorge and Ruiz, Oscar. *Interactive Visualization of Volumetric Data with WebGL in Real-time* In Proceedings of the 16th International Conference on 3D Web Technology (Web3D '11). ACM, New York, NY, USA, 137-146. `http://dx.doi.org/10.1145/2010425.2010449`
