# Supplementary figures and images for: Stochastic Simulation Service: Bridging the Gap between the Computational Expert and the Biologist
Source: PLoS Comput Biol. 2016 Dec 8;12(12):e1005220. doi: 10.1371/journal.pcbi.1005220 (PMC5145134; doi:10.1371/journal.pcbi.1005220)

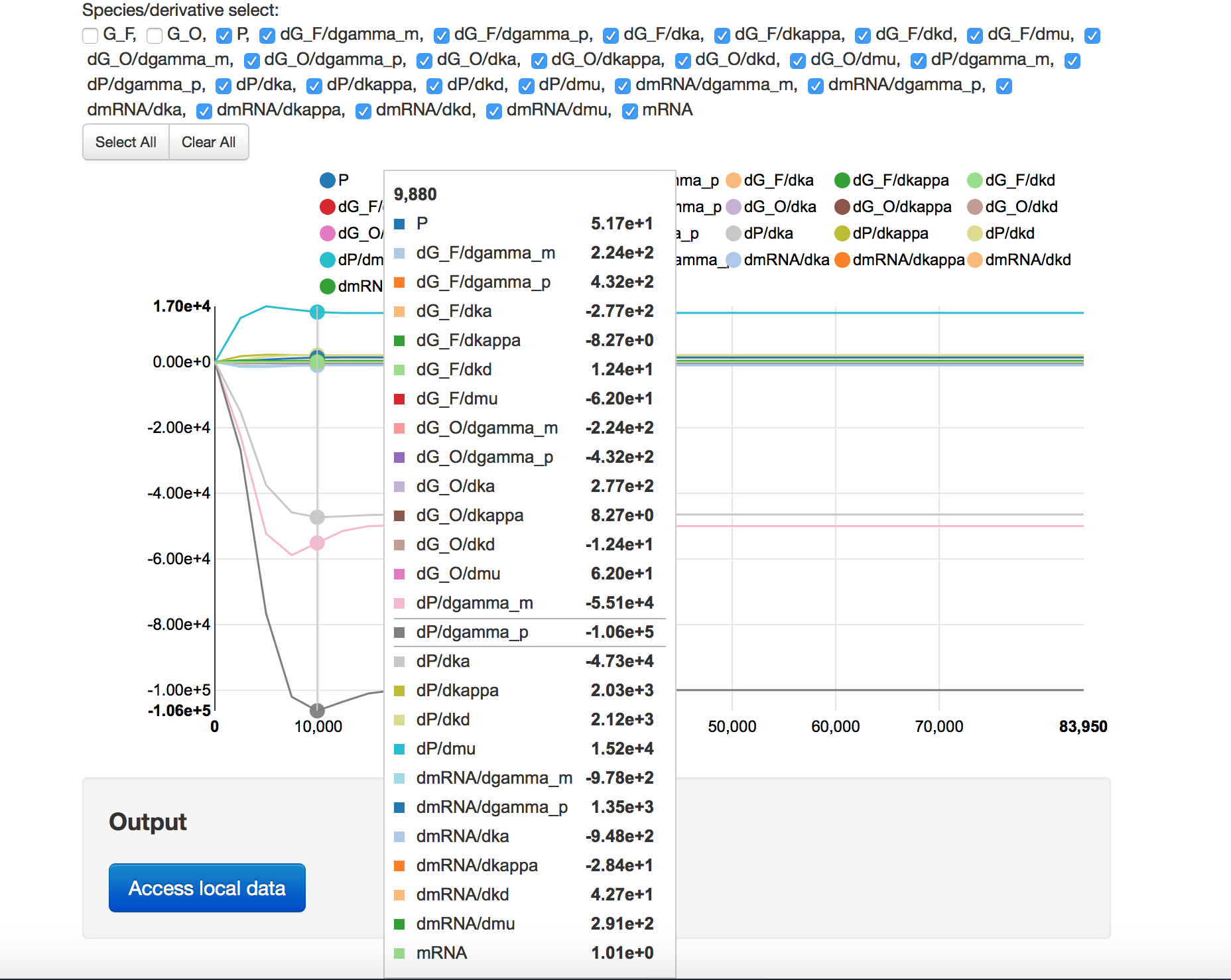

Supplement: S1 Fig — (PNG) [file pcbi.1005220.s004.png]
